# Role of Neurocellular Endoplasmic Reticulum Stress Response in Alzheimer’s Disease and Related Dementias Risk

**DOI:** 10.3390/genes15050569

**Published:** 2024-04-28

**Authors:** Miriam Aceves, Jose Granados, Ana C. Leandro, Juan Peralta, David C. Glahn, Sarah Williams-Blangero, Joanne E. Curran, John Blangero, Satish Kumar

**Affiliations:** 1 Division of Human Genetics and South Texas Diabetes and Obesity Institute, University of Texas Rio Grande Valley School of Medicine, McAllen, TX 78504, USA; miriam.aceves@utrgv.edu (M.A.); jose.granados04@utrgv.edu (J.G.); 2 Division of Human Genetics and South Texas Diabetes and Obesity Institute, University of Texas Rio Grande Valley School of Medicine, Brownsville, TX 78520, USA; ana.leandro@utrgv.edu (A.C.L.); juan.peralta@utrgv.edu (J.P.); sarah.williams-blangero@utrgv.edu (S.W.-B.); joanne.curran@utrgv.edu (J.E.C.); john.blangero@utrgv.edu (J.B.); 3 Department of Psychiatry, Boston Children’s Hospital and Harvard Medical School, Boston, MA 02115, USA; david.glahn@childrens.harvard.edu

**Keywords:** Alzheimer’s disease, dementia, iPSCs, neural stem cells, endoplasmic reticulum stress, gene expression

## Abstract

Currently, more than 55 million people around the world suffer from dementia, and Alzheimer’s Disease and Related Dementias (ADRD) accounts for nearly 60–70% of all those cases. The spread of Alzheimer’s Disease (AD) pathology and progressive neurodegeneration in the hippocampus and cerebral cortex is strongly correlated with cognitive decline in AD patients; however, the molecular underpinning of ADRD’s causality is still unclear. Studies of postmortem AD brains and animal models of AD suggest that elevated endoplasmic reticulum (ER) stress may have a role in ADRD pathology through altered neurocellular homeostasis in brain regions associated with learning and memory. To study the ER stress-associated neurocellular response and its effects on neurocellular homeostasis and neurogenesis, we modeled an ER stress challenge using thapsigargin (TG), a specific inhibitor of sarco/endoplasmic reticulum Ca^2+^ ATPase (SERCA), in the induced pluripotent stem cell (iPSC)-derived neural stem cells (NSCs) of two individuals from our Mexican American Family Study (MAFS). High-content screening and transcriptomic analysis of the control and ER stress-challenged NSCs showed that the NSCs’ ER stress response resulted in a significant decline in NSC self-renewal and an increase in apoptosis and cellular oxidative stress. A total of 2300 genes were significantly (moderated *t* statistics FDR-corrected *p*-value ≤ 0.05 and fold change absolute ≥ 2.0) differentially expressed (DE). The pathway enrichment and gene network analysis of DE genes suggests that all three unfolded protein response (UPR) pathways, protein kinase RNA-like ER kinase (PERK), activating transcription factor-6 (ATF-6), and inositol-requiring enzyme-1 (IRE1), were significantly activated and cooperatively regulated the NSCs’ transcriptional response to ER stress. Our results show that IRE1/X-box binding protein 1 (XBP1) mediated transcriptional regulation of the E2F transcription factor 1 (*E2F1*) gene, and its downstream targets have a dominant role in inducing G1/S-phase cell cycle arrest in ER stress-challenged NSCs. The ER stress-challenged NSCs also showed the activation of C/EBP homologous protein (CHOP)-mediated apoptosis and the dysregulation of synaptic plasticity and neurotransmitter homeostasis-associated genes. Overall, our results suggest that the ER stress-associated attenuation of NSC self-renewal, increased apoptosis, and dysregulated synaptic plasticity and neurotransmitter homeostasis plausibly play a role in the causation of ADRD.

## 1. Introduction

With the increasing elderly population around the world, the number of dementia cases is also rising. Currently, more than 55 million people have dementia, and the World Health Organization estimates this number will increase to 139 million by 2050. Alzheimer’s Disease and Related Dementias (ADRD) is the most common form of dementia, and accounts for 60–70% of all dementia cases [1]. Alzheimer’s disease (AD) pathology is characterized by the aggregation of extracellular senile plaques of amyloid-β (Aβ) and intracellular neurofibrillary tangles of hyperphosphorylated tau [2,3]. AD pathology has been associated with persistent neuroinflammation, neurotoxicity, and progressive neurodegeneration [4,5,6], and its spread to the brain regions associated with learning and memory is thought to cause cognitive and memory impairments in AD patients [7,8]. However, a mechanistic understanding of this causality between AD pathology and AD’s clinical symptoms remains to be established. For example, 10% to 30% of individuals who were clinically diagnosed with ADRD did not have AD neuropathology at autopsy [9] or had normal amyloid positron emission tomography (PET) and/or cerebrospinal fluid (CSF) Aβ_42_ measures (the biomarker for in vivo amyloid pathology) [10,11,12,13,14,15,16]. Similarly, 30% to 40% of elderly patients who had AD neuropathology at autopsy, or had abnormal measures of amyloid biomarkers suggestive of amyloid pathology, were not cognitively impaired [11,12,15,17,18,19]. Human and animal model data suggest a causal upstream role for amyloid/tau pathways in AD pathogenesis; and although β-amyloidosis alone may be insufficient to cause cognitive deterioration directly, it is likely to trigger a cascade of downstream pathologic changes that lead to cognitive decline [20,21,22].

Studies of the postmortem AD brain have shown elevated levels of ER stress in AD patients [23]. ER stress triggers a highly conserved signaling cascade of the unfolded protein response (UPR) that regulates cellular homeostasis by activating the protein kinase RNA-like ER kinase (PERK; also known as Eukaryotic translation initiation factor 2 α kinase 3 or EIF2AK3), activating transcription factor-6 (ATF-6), and inositol-requiring enzyme-1 (IRE1; also known as ERN1) pathways [24,25]. Accumulating evidence, however, shows that these pathways paradoxically activate a pro-apoptotic, pro-inflammatory, and oxidative stress-associated cascade of downstream events [26,27]. The UPR-induced sequestration of protein synthesis may also affect neurocellular homeostasis, synaptic plasticity, and memory consolidation functions in the brain. PERK-associated protein translation controls were shown to be linked to cognitive impairment in AD models [28,29]. Similarly, reduced synaptic protein synthesis due to the increased phosphorylation of the PERK target Eukaryotic translation initiation factor 2 α (eIF2α) caused cognitive impairment [30,31,32]. The ε4 allele of the APOE gene (APOE4) is a genetic risk factor for AD and other forms of neurodegeneration [33,34]. Although the precise mechanism by which APOE4 acts to increase AD risk remains to be understood, it has been hypothesized that APOE4 may contribute to ER and proinflammatory stresses due to its structural characteristics [35,36]. Higher levels of activating transcription factor 4 (ATF4) in the brains of APOE4 carriers and AD animal models offer further support to this hypothesis [37,38]. The existing data underscore the role of the ER stress response in neurocellular homeostasis and ADRD pathogenesis.

The presence of AD pathology and progressive neurodegeneration in the hippocampus is strongly correlated with cognitive decline in AD patients [39,40]. The hippocampus is also one of the major sites of adult neurogenesis in the brain, and accumulating evidence now suggests that adult hippocampal neurogenesis (AHN) that occurs throughout life (albeit declining with age) is essential for cellular homeostasis and hippocampus-dependent cognitive functions [41,42,43], and is severely impaired in ADRD patients [44]. The number of doublecortin (DCX)-positive immature neurons in the dentate gyrus decreases in patients with mild cognitive impairment (MCI) [43] and those in the early stages of AD, and is further reduced in later stages [44]. An ER stress-associated surge in the pro-apoptotic, pro-inflammatory, and oxidative stress processes potentially alters adult neurogenesis in the hippocampus [45] by mechanisms that are yet fully understood.

To study the ER stress-associated neurocellular response and its effects on neurocellular homeostasis and neurogenesis, we performed an ER stress challenge on the induced pluripotent stem cell (iPSC)-derived neural stem cells (NSCs) of two individuals from our Mexican American Family Study (MAFS). We have previously shown that our iPSC-derived NSCs are transcriptionally akin to the cells of the dorsal neuroepithelium, which give rise to the majority of the central nervous system, and are a relevant cell type to study developmental and adult neurogenesis [46].

## 2. Materials and Methods

Validated iPSC lines were established from the lymphoblastoid cell samples of two MAFS participants: A02287 and A09161 (both females; 58 and 59 years old, respectively, at the time of sample collection) as described in our previous publications [47,48]. The iPSCs were then differentiated into well-characterized NSCs for this study.

### 2.1. NSC Generation and Characterization

The differentiation of iPSCs into a highly uniform population of NSCs was achieved using a commercially available Gibco PSC neural induction medium (Thermo Fisher Scientific, Waltham, MA, USA) and the method described in Yan et al. [49]. Briefly, the iPSCs, maintained in feeder-free conditions, were dissociated and then seeded as 50–200 µm cell aggregates in a Geltrex (Thermo Fisher Scientific, Waltham, MA, USA)-coated tissue culture vessel and mTeSR-1 medium (Stem Cell Technologies Inc., Cambridge, MA, USA) supplemented with 10 µM ROCK inhibitor Y27632 (ATCC, Manassas, VA, USA). The iPSCs were seeded at a cell density that reached 15–25% cell confluency after 24 h when the cell culture medium was switched to Gibco PSC neural induction medium (Thermo Fisher Scientific, Waltham, MA, USA). Thereafter, the medium was changed every other day. On day 7, the differentiated NSCs were dissociated with Stem Pro Accutase (Thermo Fisher Scientific, Waltham, MA, USA) and then reseeded in a Geltrex-coated tissue culture vessel, and at a cell density of 0.1 million/cm^2^. The seeded NSCs were cultured in an NSC expansion medium containing 50% Neurobasal medium, 50% Advanced DMEM/F12, and 1X neural induction supplement (all from Thermo Fisher Scientific, Waltham, MA, USA) until confluence was reached. The NSC expansion medium was supplemented with 5 µM ROCK inhibitor Y27632 (ATCC, Manassas, VA, USA) for the first 24 h. The generated NSCs from passage one were used for characterization and all downstream ER stress challenge experiments.

The generated NSC lines were characterized by immunocytochemistry (ICC) analysis of the NSC markers Nestin (NES), Paired box 6 (PAX6), SRY-box transcription factor 2 (SOX2), and SRY-box transcription factor 1 (SOX1), and transcriptomic analysis. The ICC analysis of the NSC markers was performed using commercially available the primary antibodies mouse anti-human Nestin (MA1-110, Invitrogen, Thermo Fisher Scientific, Waltham, MA, USA), rabbit anti-human PAX6 (#60443, Cell Signaling Technology, Inc., Danvers, MA, USA), mouse anti-human SOX2 (sc-365964, Santa Cruz Biotechnology, Inc., Dallas, TX, USA), and rabbit anti-human SOX1 (#4194, Cell Signaling Technology, Inc., Danvers, MA, USA); the secondary antibodies donkey anti-mouse Alexa Fluor™ 488 and donkey anti-rabbit Alexa Fluor™ 594 (R37114 and R37119, respectively; Invitrogen, Thermo Fisher Scientific, Waltham, MA, USA); and standard ICC techniques.

### 2.2. ER Stress Challenge Assay

After the ICC and transcriptomic characterization, the generated NSCs were seeded on the Geltrex-coated surface and maintained in the NSC expansion medium (described above) until 60–70% confluence was reached. One part of the NSC culture was then vehicle-treated (control) and the other two parts were ER stress-challenged using two different concentrations, 0.3 µM and 0.6 µM, of thapsigargin (TG) for 24 h. TG is a specific inhibitor of sarco/endoplasmic reticulum Ca^2+^ ATPase (SERCA) and is a classical tool commonly used to study ER stress and UPR biology in mammalian cells. Both vehicle-treated and ER stress-challenged NSCs were then quantitatively measured for cell proliferation (quantified as post-assay total live cell count minus the total number of cells seeded), apoptotic cell death, protein aggregation, oxidative stress, and mitochondrial function cellular phenotypes by high-content screening (HCS) analysis, and for genome-wide gene expression using deep mRNA sequencing.

### 2.3. Analysis of Cellular Phenotypes

Total and live cell counts were performed using 0.4% trypan blue staining (Invitrogen, Thermo Fisher Scientific, Waltham, MA, USA) and a CellDrop automated cell counter (DeNovix Inc., Wilmington, DE, USA). Intracellular aggregates of the unfolded protein (Amyloid) were stained with Thioflavin T (Thermo Scientific Chemicals, Thermo Fisher Scientific, Waltham, MA, USA). Generalized oxidative stress was assessed by staining the cells with a CellROX Green fluorogenic probe and a ThiolTracker™ Violet intracellular thiol probe (Invitrogen, Thermo Fisher Scientific, Waltham, MA, USA). MitoTracker™ Red CMXRos dye was used to stain functional mitochondria in the cells. Apoptotic cells were stained using CellEvent™ Caspase-3/7 Green ReadyProbes™ reagents (Invitrogen, Thermo Fisher Scientific, Waltham, MA, USA). The staining procedures were performed on live cells and following the manufacturer’s instructions. Cell nuclei were counterstained with Hoechst 33342 (Invitrogen, Thermo Fisher Scientific, Waltham, MA, USA), and appropriate negative controls were included. Immediately after staining, cells were imaged live on a PerkinElmer Operetta high-content screening system (PerkinElmer, Waltham, MA, USA) and quantifications of cellular phenotypes were performed using Harmony software v4.1 (PerkinElmer, Waltham, MA, USA).

### 2.4. RNA Extraction and Sequencing

Total RNA from both control and ER stress-challenged NSCs was extracted using a commercially available RNeasy Mini Kit (Qiagen, Germantown, MD, USA) and by following the manufacturer’s instructions. The quality and quantity of the extracted RNA were measured using a NanoDrop 2000 Spectrophotometer (Thermo Fisher Scientific, Waltham, MA, USA) and an Agilent 2200 TapeStation system (Agilent, Santa Clara, CA, USA).

RNA sequencing was performed on an Illumina NovaSeq6000 instrument using the Illumina Stranded mRNA Prep Ligation Kit. Briefly, mRNA sequencing libraries were generated from 1 μg high-quality total RNA from each sample using the reagents supplied in Illumina Stranded mRNA sample preparation kit v2 (Illumina, Inc., San Diego, CA, USA). First, poly-A-tailed mRNAs were enriched from the total RNA samples using oligo-dT magnetic beads supplied in the kit. The enriched mRNAs were then fragmented into ~200–600 base-pair-sized molecules using divalent cations and elevated temperature. The first-strand cDNA was synthesized from the fragmented RNA by reverse transcription and using random primers, followed by second-strand cDNA synthesis using DNA polymerase-I and RNase H. The generated cDNA fragments were end-repaired, and adaptor ligations were performed. The resulting cDNA libraries were then purified, enriched by polymerase chain reaction, and deep-sequenced on an Illumina NovaSeq6000 instrument.

### 2.5. RNA Sequencing and Differential Gene Expression Analysis

The Illumina bcl2fastq2 conversion software v2.20 (Illumina, Inc., San Diego, CA, USA) was used to demultiplex sequencing data and convert base call files generated by the sequencing system into raw fastq files. Pre-alignment quality controls and sequence alignments were performed using StrandNGS software v4.1 (Strand Life Sciences Pvt. Ltd., Bangalore, India). The sequences were aligned to human genome assembly GRCh38 (hg38) and mapped to RefSeq transcripts. The aligned reads were filtered based on read quality metrics, and then, log transformation and “DESeq” normalization were performed. Only known genes/mRNAs with a normalized read count (NRC) ≥ 10 were analyzed for differential gene expression as per the conditions described in the results.

To identify significantly differentially expressed genes/mRNAs, moderated *t* statistics and expression fold change (FC) analyses were performed. The genes with a moderated *t* statistics FDR-corrected *p*-value ≤ 0.05 and FC absolute (FC abs) ≥ 2.0 between the pair(s) of conditions as described in the results were considered significantly differentially expressed (DE).

### 2.6. Functional Enrichment and Pathway Analysis

Functional annotations and pathway enrichment analyses of DE genes were performed using the Ingenuity Pathway Analysis (IPA) platform (QIAGEN Digital Insights, Redwood City, CA, USA), Kyoto Encyclopedia of Genes and Genomes (KEGG) pathway database, and ‘Enrichr’ and ‘ShinyGO v0.77’ gene set enrichment web tools [50,51] (accessed February 2024). In IPA, enrichment significance was determined by right-tailed Fisher’s exact test FDR-corrected *p*-values, and the direction of functional change was assessed by the activation z-score as described in Kramer et al. (2014) [35]. Briefly, the activation states of an enriched biological function were inferred using the z-score. A positive z-score indicates activation and a negative z-score indicates inhibition of the biological function. The inference was based on relationship between genes and biological function(s) that were literature-derived. The direction of effect (activation or inhibition) was determined by DE genes and the direction of the gene’s effects on the biological function. To map the identified gene sets to KEGG pathways, KEGG mapper v5, as detailed in Kanehisa et al., 2022 [52,53], was used. Several enrichment scores are implemented in ‘Enrichr’, which are described in Chen et al. (2013) [50]; we ranked our ‘Enrichr’ results based on the Fisher’s exact test *p*-values. In ‘ShinyGO’ enrichment analyses, statistical significance was assessed using FDR-corrected *p*-values (≤0.05).

## 3. Results

### 3.1. Validation of iPSC-Derived NSCs

**Figure 1:**
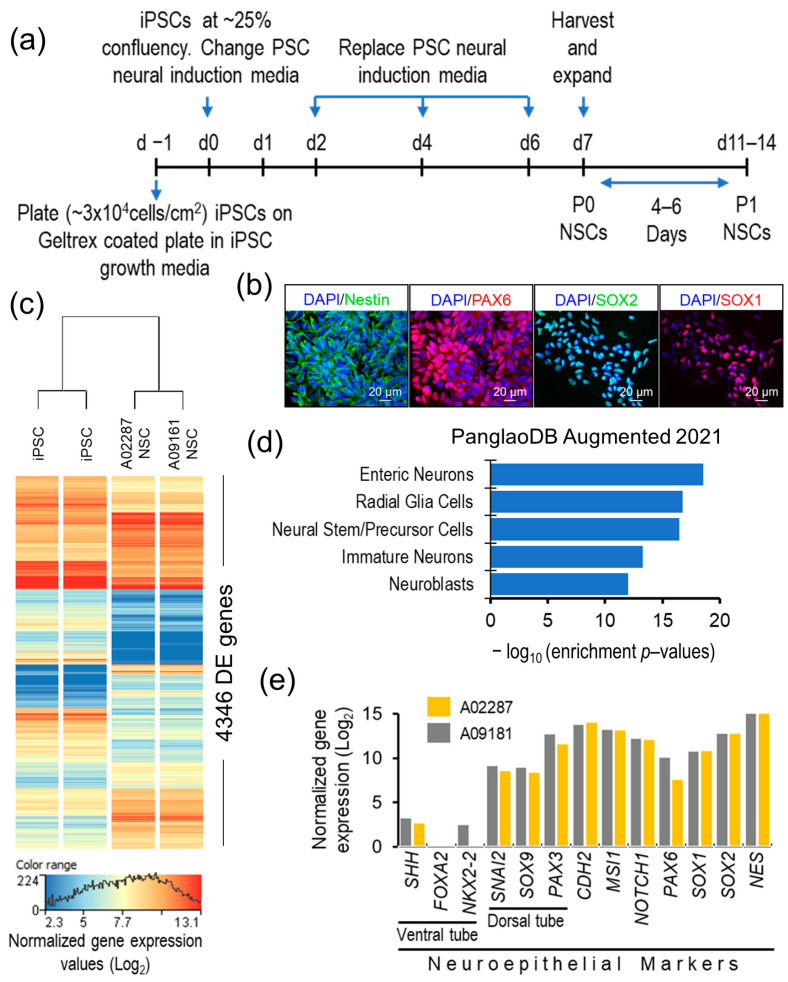
Validation of generated NSCs. (**a**) A schematic outline of the NSC differentiation method. (**b**) Representative ICC images showing expression of NSC markers Nestin, PAX6, SOX2, and SOX1 in generated NSCs. (**c**) Heap map of transcriptome-wide differentially expressed genes between iPSCs and their differentiated NSCs. (**d**) Cell type gene set (PanglaoDB Augmented 2021) enrichment analysis plot of the generated NSCs’ upregulated transcriptome. (**e**) Bar plot showing dorsal neuroepithelial gene expression profile of the generated NSCs.

The differentiation of the iPSC lines A02287 and A09161, which were established from the samples of two MAFS participants, into NSCs was carried out using the protocol described in the methods and summarized in Figure 1a.

Both generated NSC lines expressed the NSC-specific markers NES, PAX6, SOX2, and SOX1 (Figure 1b), and showed large-scale but uniform resetting of the expressed transcriptome during differentiation. A total of 4346 genes were significantly DE (moderated *t* statistics *p*-value ≤ 0.05, FC abs ≥ 2.0) between iPSCs and the differentiated NSCs (Figure 1c). The 2113 genes that were significantly upregulated in the generated NSCs showed the top five significant enrichment (*p*-value ranged from 2.87 × 10^−19^ to 1.03 × 10^−12^) in the PanglaoDB gene set for enteric neurons, radial glia cells, neural stem/precursor cells, immature neurons, and neuroblasts (Figure 1d). High expression of the neuroepithelial genes *NES*, *SOX2*, *SOX1*, *PAX6*, Notch receptor 1 (*NOTCH1*), Musashi RNA-binding protein 1 (*MSI1*), and Chromodomain helicase DNA-binding protein 2 (*CHD2*), and the genes of the dorsal tube neuroepithelium Paired box 3 (*PAX3*), Growth differentiation factor 7 (*GDF7*), SRY-box transcription factor 9 (*SOX9*), and Snail family transcriptional repressor 2 (*SNAI2*), and a lack of or very low expression of ventral tube neuroepithelial markers, NK2 homeobox 2 (*NKX2-2*), Forkhead box A2 (*FOXA2*), and Sonic hedgehog signaling molecule (*SHH*), suggest a dorsal tube neuroepithelial transcriptomic and functional profile of the generated cells (Figure 1e). Furthermore, in our previous publication [46], we demonstrated that the generated NSCs possess the apical–basal polarity characteristics of the neuroepithelium. These results validate the NSC characteristics of the generated cells and their potential as in vitro cell models of human neuropsychiatric and neurodegenerative disease, particularly to study neural cell homeostasis in the brain.

### 3.2. Effect of ER Stress on Neurocellular Homeostasis

**Figure 2:**
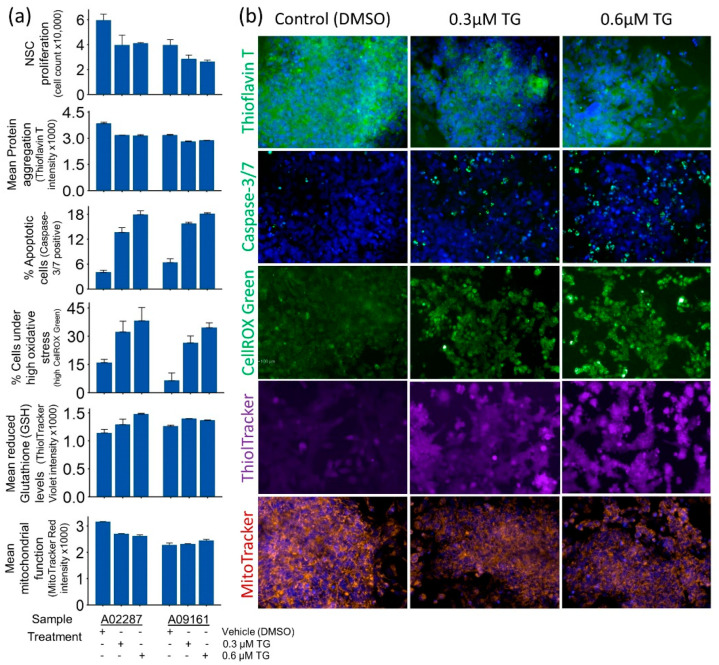
Quantitative measures of cellular homeostasis in control and ER stress-challenged NSCs. (**a**) Bar plots of cell proliferation, protein aggregation, apoptosis, generalized oxidative stress, reduced glutathione (GSH), and functional mitochondria in vehicle-treated controls and ER stress-challenged NSCs. (**b**) Representative cytochemistry images of cellular phenotypes quantified in panel (**a**). All images were obtained using a 20× long-working-distance objective on a PerkinElmer Operetta high-content screening system.

Pharmacologically induced ER stress using 0.3 µM and 0.6 µM TG for 24 h showed a dose-independent effect on NSC homeostasis. The mean protein (amyloid) levels per cell measured by Thioflavin T staining and HCS analysis decreased in ER stress-challenged NSCs (Figure 2a,b).

ER stress-induced UPR plausibly attenuated new protein synthesis and accelerated the de-aggregation of proteins, leading to decreased protein (amyloid) levels in ER stress-challenged NSCs. Interestingly, a significant decline in cell proliferation and increased apoptosis and cellular oxidative stress were also observed in ER stress-challenged NSCs (Figure 2a,b). The change in NSCs’ mitochondrial function, however, was minimally affected and showed mixed results in the two samples.

### 3.3. NSCs’ Transcriptomic Response to ER Stress

**Figure 3:**
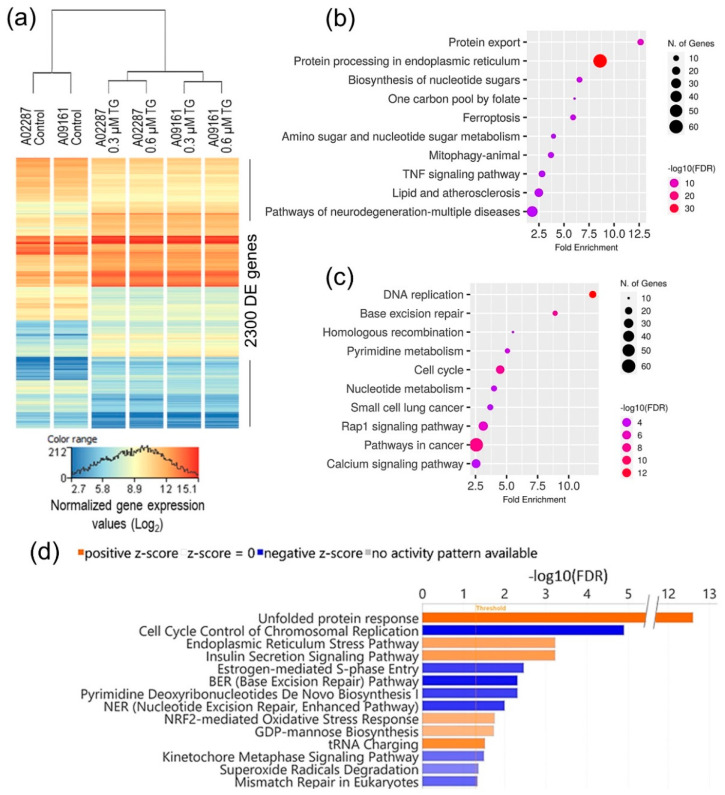
Transcriptomic analysis of NSCs’ ER stress response. (**a**) Heatmap of 2300 genes, which were significantly differentially expressed between control and ER stress-challenged NSCs. (**b**,**c**) Top 10 KEGG pathways, which were significantly enriched in upregulated and downregulated DE genes, respectively. (**d**) IPA canonical pathways, which were significantly enriched (FDR-corrected *p*-value ≤ 0.05), and differentially regulated (activation absolute z-score ≥ 2.0) in ER stress-challenged NSCs.

To investigate the mechanistic relationship between ER stress and the observed change in NSC homeostasis, we performed a genome-wide differential gene expression analysis and identified 2300 genes, which were significantly DE (moderated *t* statistics FDR-corrected *p*-value ≤ 0.05 and FC abs ≥ 2.0) between the control and ER stress-challenged NSCs (Figure 3a; and Table S1).

The NSCs’ transcriptomic response to ER stress was independent of the TG dose used (Figure 3a), and suggests significant activation of the protein processing, protein export, and unfolded protein response pathways, and the inhibition/downregulation of DNA and chromosomal replication and cell cycle-related pathways (Figure 3b–d). Intriguingly, pathways of neurodegeneration in multiple diseases were among the top 10 KEGG pathways enriched in the upregulated DE genes (Figure 3b).

### 3.4. Unfolded Protein Response

**Figure 4:**
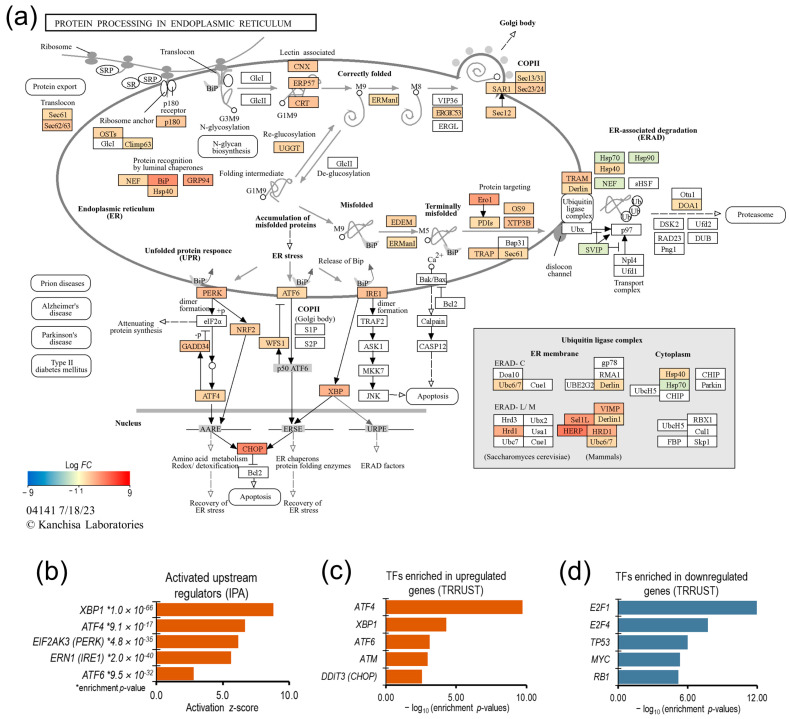
Gene enrichment analysis of NSCs’ unfolded protein response. (**a**) KEGG pathway map for “protein processing in endoplasmic reticulum” showing transcriptional upregulation of UPR-associated genes in ER stress-challenged NSCs. (**b**) Bar plot of upstream regulators, which were predicted to be significantly activated and enriched in the DE genes. (**c**,**d**) Bar plots of transcription factors, which were significantly enriched in significantly up- and downregulated genes, respectively.

ER stress induced a strong UPR in NSCs: the genes associated with protein export, BiP (binding immunoglobulin protein)-mediated protein degradation, and all three UPR pathways (PERK, ATF6, and IRE1) were significantly upregulated (Figure 4). The gene expression of ER luminal chaperones, Heat shock protein family A (Hsp70) member 5 (*HSPA5*; also known as BiP/GRP78) and Heat shock protein 90 β family member 1 (*HSP90B1*; also known as GRP94), which play important roles in the UPR activation and ER-associated degradation (ERAD) of unfolded proteins, was upregulated more than 16- and 9-fold, respectively.

The upstream regulator analysis of the DE genes using the IPA knowledge base, as well as the enrichment analysis of DE genes in the TRRUST v2 (Transcriptional Regulatory Relationships Unraveled by Sentence-based Text mining) database using the ‘Enrichr’ web tool, overwhelmingly suggest that the NSCs’ transcriptional response to ER stress was predominantly regulated by UPR pathways (Figure 4b–d). Therefore, we focused the rest of our investigation on better understanding the transcriptional regulation downstream of the UPR pathways in NSCs.

### 3.5. UPR Effectors IRE1/XBP1, PERK/ATF4/NRF2, and ATF6 Cooperatively Regulate the Transcription of ER Protein-Processing Genes

**Figure 5:**
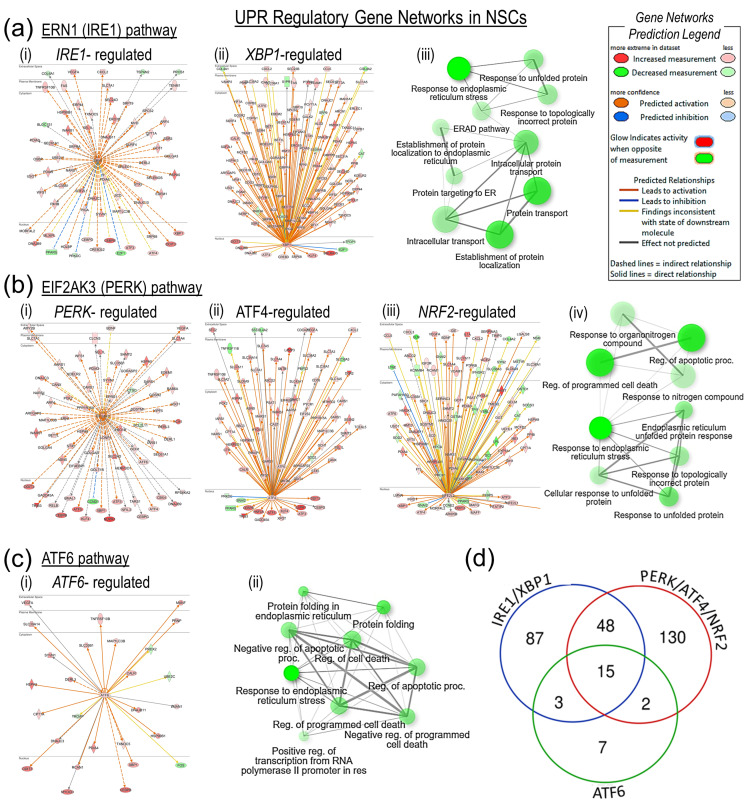
UPR regulatory gene networks in NSCs’ ER stress response. (**a-i**,**a-ii**) *IRE1* and *XBP1* gene networks show their direct downstream DE targets. (**a-iii**) Functional gene ontology (GO) term enrichment analysis of the DE target genes identified by *IRE1* and *XBP1* gene network analysis. (**b-i**,**b-ii,b-iii**) *PERK*, *ATF4*, and *NRF2* gene networks and their direct downstream DE targets. (**b-iv**) Functional enrichment analysis of DE target genes identified by *PERK*, *ATF4*, and *NRF2* gene network analysis. (**c-i**) *ATF6* gene network showing its direct downstream DE targets. (**c-ii**) Functional enrichment analysis of DE target genes identified by *ATF6* gene network analysis. (**d**) Venn diagram showing number of DE genes that were common downstream targets of IRE1/XBP1, PERK/ATF4/NRF2, and ATF6 pathways.

To identify transcriptomic effects that were directly downstream of the UPR effector kinases and transcription factors, we performed upstream regulator and gene network enrichment analysis of the DE genes in IPA. The 153 DE genes that were predicted to be direct downstream targets of the IRE1/XBP1 pathway showed significant enrichment (the FDR-corrected *p*-value ranged from 1.1 × 10^−39^ to 6.2 × 10^−24^) in response to ER stress, protein transport, protein localization, and the response to unfolded protein-associated gene GO biological processes (Figure 5a). Interestingly, most IRE1/XBP1-predicted downstream targets were significantly upregulated, except for a few notable transcription factors, i.e., E2F transcription factor 1 (*E2F1*), Transcription factor Dp-1 (*TFDP1*), and Peroxisome proliferator-activated receptor γ (*PPARG*). The 195 DE genes that were the predicted targets of the PERK/ATF4/NFE2-like bZIP transcription factor 2 (NFE2L2; also known as NRF2) pathway were significantly enriched (the FDR-corrected *p*-value ranged from 9.3 × 10^−29^ to 1.4 × 10^−17^) in response to ER stress, apoptosis, and the unfolded protein response-associated GO biological processes and were significantly upregulated (Figure 5b). A few transcription factor genes that were significantly downregulated included PERK target Cyclin D1 (*CCND1*); the ATF4 targets *PPARG*, *SNAI2*; and protein kinase, DNA-activated, catalytic subunit (*PRKDC*), and the NRF2 targets Cyclin E2 (*CCNE2*), *PPARG*, *SNAI2*, and FKBP prolyl isomerase 5 (*FKBP5*). The 27 ATF6 downstream DE targets were significantly enriched (the FDR-corrected *p*-value ranged from 7.6 × 10^−10^ to 1.8 × 10^−05^) in response to ER stress, protein folding, apoptotic cell death, and protein degradation GO processes (Figure 5c). The C/EBP homologous protein gene (*CHOP*; also known as DNA damage-inducible transcript 3 or *DDIT3*), which is the downstream target of UPR pathways, was significantly upregulated. Functional enrichment analysis of CHOP’s downstream targets (Figure S1) showed the activation of ‘intrinsic apoptotic signaling’ and ‘positive regulation of transcription from RNA polymerase II promoter’ GO biological processes. Overall, there was a substantial overlap in the IRE1/XBP1, PERK/ATF4/NRF2, and ATF6 downstream DE target genes (Figure 5d), as well as in the enriched GO biological processes, which suggests that UPR pathways cooperatively regulate NSCs’ transcriptional response to ER stress.

### 3.6. The IRE1/XBP1 Pathway Induces Cell Cycle Arrest through E2F1

**Figure 6:**
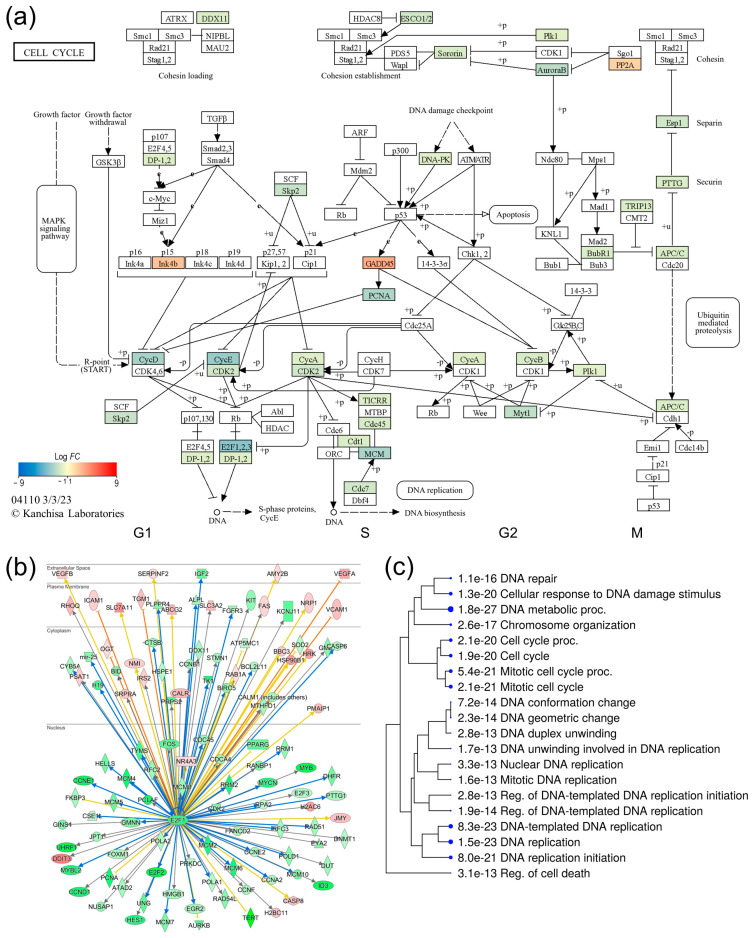
Transcriptional regulation of cell cycle in ER stress-challenged NSCs. (**a**) KEGG ‘cell cycle’ pathway map showing ER stress-induced transcriptional inhibition of cell cycle progression. (**b**) *E2F1* gene network showing its direct downstream DE targets. (**c**) GO biological process enrichment analysis of the *E2F1* DE targets.

Whilst the majority of the DE genes that were immediately downstream of the UPR effectors IRE1, XBP1, PERK, ATF4, NRF2, and ATF6 were significantly upregulated (Figure 5), nearly half of the post-ER-stress DE genes (1169 genes) were significantly downregulated (Table S1). The functional enrichment analysis of the downregulated genes suggested a significant inhibition of cell cycle progression in ER stress-challenged NSCs (Figure 3c,d). The mapping of DE genes to the cell cycle KEGG pathway (Figure 6a) shows that out of 37 DE genes that were mapped, only three genes, Growth arrest and DNA damage-inducible α (*GADD45A*), Cyclin-dependent kinase inhibitor 2B (*CDKN2B*), and Protein phosphatase 2 regulatory subunit B′β (*PPP2R5B*), were upregulated and are known for their negative control of cell growth and cell division. The remaining 34 genes that were mapped to the cell cycle pathway were all significantly downregulated, and included the cyclin genes *CCND1*, Cyclin A2 (*CCNA2*), Cyclin B1 (*CCNB1*), Cyclin E1 (*CCNE1*), and *CCNE2*; Cyclin-dependent kinase 2 (*CDK2*)*;* the E2F transcription factors *E2F1*, *E2F2*, and *E2F3*; the DNA replication licensing factors Chromatin licensing and DNA replication factor 1 (*CDT1*), Cell division cycle 7 (CDC7), and Cell division cycle 45 (*CDC45*); and the Minichromosome maintenance (MCM) complex component genes, *MCM2*, *MCM3*, *MCM4*, *MCM5*, *MCM6*, and *MCM7*. Overall, these results suggest G1/S-phase cell cycle arrest in ER stress-challenged NSCs (Figure 6a). The upstream regulator and gene network analysis of DE genes using the IPA knowledge base suggests that the transcription factor *E2F1*, which is a downstream target of XBP1, regulates the transcription of cell cycle progression genes, including the other E2Fs, cyclin genes, the *CDK2* gene, DNA replication licensing factors, and MCM complex genes (Figure 6b).

### 3.7. UPR Transcriptional Attenuation Affects Synaptic Plasticity and Neurotransmitter Homeostasis

**Figure 7:**
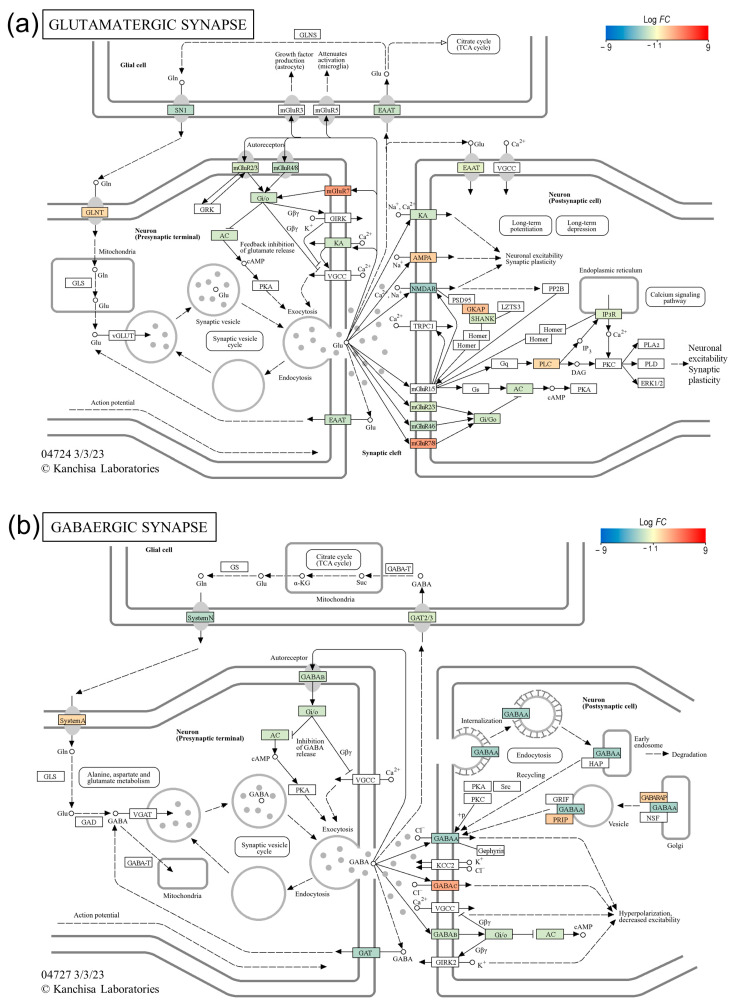
ER stress-induced transcriptional changes in glutamatergic and GABAergic synapses. (**a**) KEGG ‘glutamatergic synapse’ pathway map showing dysregulated transcription of ionotropic glutamate receptors (iGluRs), metabotropic glutamate receptors (mGluRs), and glutamate-transporter EAATs genes in ER stress-challenged NSCs. (**b**) KEGG ‘GABAergic synapse’ pathway map showing dysregulated transcription of GABA receptors and GABA-transporter GATs genes in ER stress-challenged NSCs.

Next, we sought to investigate the effect of ER stress-induced transcriptional changes on synaptic plasticity and neurotransmitter homeostasis, the neurocellular function important to learning and memory. As shown in the glutamatergic and GABAergic synapse pathway maps in Figure 7, ER stress resulted in the transcriptional dysregulation of neurotransmitter receptors and transporter genes in both types of synapses, which plausibly affects synaptic plasticity and neurotransmitter homeostasis, which are important for learning and memory functions. In the glutamatergic synapse, the expression of the genes Glutamate ionotropic receptor NMDA-type subunit 2A (*GRIN2A*) and Glutamate ionotropic receptor kainate-type subunit 1 (*GRIK1*), which encode the ionotropic glutamate receptors (iGluRs) N-methyl-D-aspartate receptor (NMDAR) and kainate-type receptor (KA), was significantly downregulated; however, the expression of the α-amino-3-hydroxy-5-methyl-4-isoxazole propionic acid-type receptor (AMPAR) gene Glutamate ionotropic receptor AMPA-type subunit 3 (*GRIA3*) was significantly upregulated. Similarly, the expression of metabotropic glutamate receptor (mGluR) genes (*GRM4* and *GRM2*) was downregulated, and the expression of *GRM7* was upregulated. The expression of genes encoding glutamate-transporter EAATs (Solute carrier family 1 member 2 or *SLC1A2*, and Solute carrier family 1 member 3 or *SLC1A3*) was also downregulated. In the GABAergic synapse expression of genes, the genes that encode γ-aminobutyric acid (GABA) receptors (GABAA and GABAB) and GABA-transporter GATs (Solute carrier family 6 member 1 or *SLC6A1* and Solute carrier family 6 member 11 or *SLC6A11*) were significantly downregulated, whereas the expression of the gene for the GABAC receptor was upregulated (Figure 7).

## 4. Discussion

Although the etiology of ADRD is yet to be fully understood, the spread of AD pathology and progressive neurodegeneration in the hippocampus is strongly correlated with cognitive decline in AD patients [39,40]. Furthermore, AD animal models and studies of postmortem AD brains suggest that elevated ER stress may have a role in ADRD pathogenesis, plausibly through altered neurocellular homeostasis in the hippocampus [23,28,29,45]. The increased levels of BiP, pPERK, pIRE1α, peIF2 α, and ATF4 in AD brains suggest chronic UPR activation [54,55,56]. However, the extent of UPR activation and its downstream effects in AD brains has not been fully characterized [57]. In this study, we modeled ER stress in iPSC-derived NSCs to investigate the neurocellular ER stress response and its role in neurocellular homeostasis. We have shown that we achieved highly uniform NSC differentiation from the iPSCs of two different individuals, and the generated NSCs possess transcriptomic and functional profiles akin to those of the cells of the dorsal neuroepithelium (Figure 1), a cell type that gives rise to the entire nervous system during development and is a relevant cell model to study neurogenesis and neurocellular homeostasis [46,58]. Modeling ER stress elicited a strong response in NSCs, resulting in a significant decline in NSC self-renewal/proliferation, and increased apoptosis and cellular oxidative stress (Figure 2). Genome-wide differential gene expression analysis to understand the mechanistic relationship between ER stress and neurocellular homeostasis showed the anticipated upregulation of protein processing in ER and UPR-associated genes (Figure 3). Interestingly, however, genes that were downregulated post-ER stress challenge suggest the transcriptional regulation of the cell cycle in ER stress-challenged NSCs (Figure 6a). Contrary to previous reports that the PERK-dependent phosphorylation of eIF2α, and the resulting global sequestration of protein synthesis, inhibit cyclin D1 translation and induce cell-cycle arrest [59], our results strongly suggest that transcriptional regulation through transcription factor E2F1, which is a downstream target of IRE1/XBP1, may play a significant, if not a dominant, role in ER stress-induced G1/S-phase cell cycle arrest (Figure 6b,c). The transcriptional repression of *E2F1* by the IRE1/XBP1 and ATF6 UPR pathways and its pro-apoptotic role, along with the upregulated expression of the ATF4 pro-apoptotic targets BCL2 binding component 3 (*BBC3*; also known as Puma) and Phorbol-12-myristate-13-acetate-induced protein 1 (*PMAIP1*; also known as Noxa), during ER stress has been reported previously [60]. E2F1 knockout murine embryonic fibroblasts, however, were reported to be resistant to ER stress-induced apoptosis [61]. Furthermore, it has been suggested that the transcriptional upregulation of the XBP1 target *E2F7* and the protein activation of ATF6 cooperatively suppress *E2F1* transcription [60,62]. However, despite *E2F7* being robustly expressed in NSCs, we did not see any significant change in its expression in the ER stress-challenged NSCs. In our data set, the expression of *E2F1* and its downstream cell cycle progression genes, including the cyclins, *CDK2* gene, DNA replication licensing factors, and MCM complex genes, was significantly downregulated. While the expression of *CCND1* was significantly downregulated, no significant change in the expression of *CDK4/6* and *RB1*, though robustly expressed in NSCs, was observed. Furthermore, the expression of *CCNE1*, *CCNE2*, and *CDK2* was significantly downregulated (Figure 6b; Table S1). These results suggest that the ER stress-induced transcriptional inhibition of *E2F1* and its downstream targets leads to cell cycle arrest at the G1-to-S phase progression ‘restriction point’ (Figure 6). During a normal cell cycle, cyclin D and the CDK4/6 complex phosphorylate the protein RB (encoded by the *RB1* gene). The unphosphorylated RB represses the expression of E2Fs and their regulated genes to inhibit the G1-to-S phase transition. The phosphorylation of RB allows for the transcription of E2Fs and their downstream targets cyclin E, cyclin A, and cyclin B. Cyclin E binds and activates CDK2, and the cyclin E/CDK2 active complex phosphorylates a broad range of proteins involved in cell cycle progression, DNA replication, chromosomal duplication, and histone modifications [63,64,65,66]. Furthermore, the significant downregulation of the cyclin gene (*CCNA2*), DNA replication licensing factors (*CDT1*, CDC7, and *CDC45*), and MCM complex genes (*MCM2*, *MCM3*, *MCM4*, *MCM5*, *MCM6*, and *MCM7*) suggests the inhibition of the S-phase DNA and chromosomal replication processes in ER stress-challenged NSCs. Due to the short G1 phase, activated NSCs and other progenitors rely on the higher expression of DNA replication licensing factors, and MCM complexes, for timely DNA duplication during the cell cycle [67,68,69].

As stated above, significant apoptosis was observed in the ER stress-challenged NSCs (Figure 2). The expression of transcription factor *DDIT3* (CHOP), which regulates the expression of a number of anti- and pro- apoptotic genes and is the downstream target of UPR pathways [70], was significantly upregulated in post-ER-stress NSCs. Functional enrichment analysis of CHOP’s downstream targets showed the upregulation of the intrinsic apoptotic signaling genes *BBC3*, Tribbles pseudokinase 3 (*TRIB3*), *ATF4*, TNF receptor superfamily member 10b (*TNFRSF10B*), and Protein phosphatase 1 regulatory subunit 15A (*PPP1R15A*; also known as *GADD34*). The upstream regulator and gene network analysis also showed that CHOP plays a role in the transcriptional regulation of several genes, including its own and some of its upstream UPR regulators (Figure S1), suggesting plausible feedback loops that may play a role in fine-tuning life/death cell fate decisions. Such feedback loops between ATF4, GADD34, and CHOP have been reported recently, where the CHOP downstream target GADD34 has been suggested to have a positive effect on ATF4 activity, while CHOP inhibits it [71,72]. Our data overwhelmingly suggest that *E2F1* downregulation leads to ER stress-induced cell cycle arrest. We further explored whether *E2F1* downregulation, as suggested previously [60,62], also has a role in ER stress-induced apoptosis in NSCs. Interestingly, genes that were predicted to be the downstream targets of *E2F1*, and whose expression was significantly upregulated in ER stress-challenged NSCs (Figure 6b), showed the highest enrichment (*p*-value = 5.9 × 10^−8^) in the KEGG apoptosis pathway, as well as positive regulation of the apoptotic signaling pathway and the positive regulation of apoptotic process GO terms ((*p*-value = 2.2 × 10^−6^ and 2.6 × 10^−6^, respectively), and included the Harakiri BCL2-interacting protein (*HRK*), Caspase 8 (*CASP8*), *DDIT3*, Phorbol-12-myristate-13-acetate-induced protein 1 (*PMAIP1*), Fas cell surface death receptor (*FAS*), and *BBC3* genes. However, several of the relationships between pro-apoptotic genes and *E2F1* were predicted to be contrary to the expected effect (represented by yellow lines in Figure 6b), suggesting that this is an area that needs further investigation.

Our results also show that ER stress-induced transcriptional changes may also affect synaptic plasticity and neurotransmitter homeostasis, processes important in learning and memory. In the ER stress-challenged NSCs, the expression of genes that encode NMDA and KA receptors was significantly downregulated, and the expression of the AMPA receptor gene was significantly upregulated, which suggests that this dysregulation of iGluRs may affect postsynaptic Ca^2+^ homeostasis in the glutamatergic synapse (Figure 7a). Postsynaptic rises in Ca^2+^ and the subsequent activation of downstream signaling molecules are crucial for long-term forms of synaptic plasticity in the hippocampus, including long-term potentiation (LTP) and long-term depression (LTD) [73,74]. Similarly, the genes encoding the GABAA and GABAB receptors were also significantly downregulated in the ER stress-challenged NSCs. GABA neurotransmission is primarily mediated by ionotropic (GABAA) and metabotropic (GABAB) receptors [75,76] and plays an important role in the regulation of hippocampal neural network activity involved in memory [77]. Moreover, the significant downregulation of glutamate-transporter EAATs and GABA-transporter GATs suggests that ER stress may also affect neurotransmitter homeostasis.

## 5. Conclusions

Overall, our ER stress modeling in iPSC-derived NSCs shows that the ER stress response leads to a significant decline in NSC self-renewal and an increase in apoptosis and cellular oxidative stress. These ER stress-induced alterations in NSCs suggest that ER stress plausibly affects neurocellular homeostasis in the brain and has a role in the causation of ADRD. We report a comprehensive list of 2300 genes that constituted NSCs’ ER stress response and suggest a significant role of transcriptional regulation in the UPR. To the best of our knowledge, we show for the first time that the IRE1/XBP1 transcriptional regulation of *E2F1* and its downstream targets plays a major role in cell cycle regulation and thus in ER stress-induced alteration in neurocellular homeostasis. The IRE1/XBP1-mediated inhibition of *E2F1* transcription caused G1/S-phase cell cycle arrest in NSCs. Intriguingly, our results also suggest that ER stress-induced transcriptional changes affect synaptic plasticity and neurotransmitter homeostasis, which may have a direct role in the causation of ADRD. There are a few important caveats to our study. We studied the ER stress response only in vitro in iPSC-derived NSCs, which are a relevant cell type to study neurocellular homeostasis, and are close surrogates for primary neural stem/progenitor cells; however, mature neurons and other cell types in the brain may have an ER stress response that is different from NSCs and may also confound the NSC response. The iPSC-derived cells are minimally influenced by life-experienced environmental exposures, an important factor in the causation of ADRD, an important factor in the causation of ADRD. Lastly, we did not have ADRD data on individuals whose samples were used for iPSC derivation.

## Data Availability

The mRNA sequence data generated from the two control and four ER stress-challenged (two replicates/line) iPSC-derived NSC lines (*n* = 6) were submitted to the gene expression omnibus (GEO) archive and are available under the accession number GSE263319.

## Supplementary Materials

The following supporting information can be downloaded at: https://www.mdpi.com/article/10.3390/genes15050569/s1, Table S1: Genes that were significantly differentially (DE) expressed between control (vehicle-treated) and ER stress-challenged NSCs. Figure S1: *DDIT3 (CHOP)* gene network showing its direct downstream DE targets.
