# Supplementary figures and images for: Role of Neurocellular Endoplasmic Reticulum Stress Response in Alzheimer’s Disease and Related Dementias Risk
Source: Genes (Basel). 2024 Apr 28;15(5):569. doi: 10.3390/genes15050569 (PMC11121587; doi:10.3390/genes15050569)

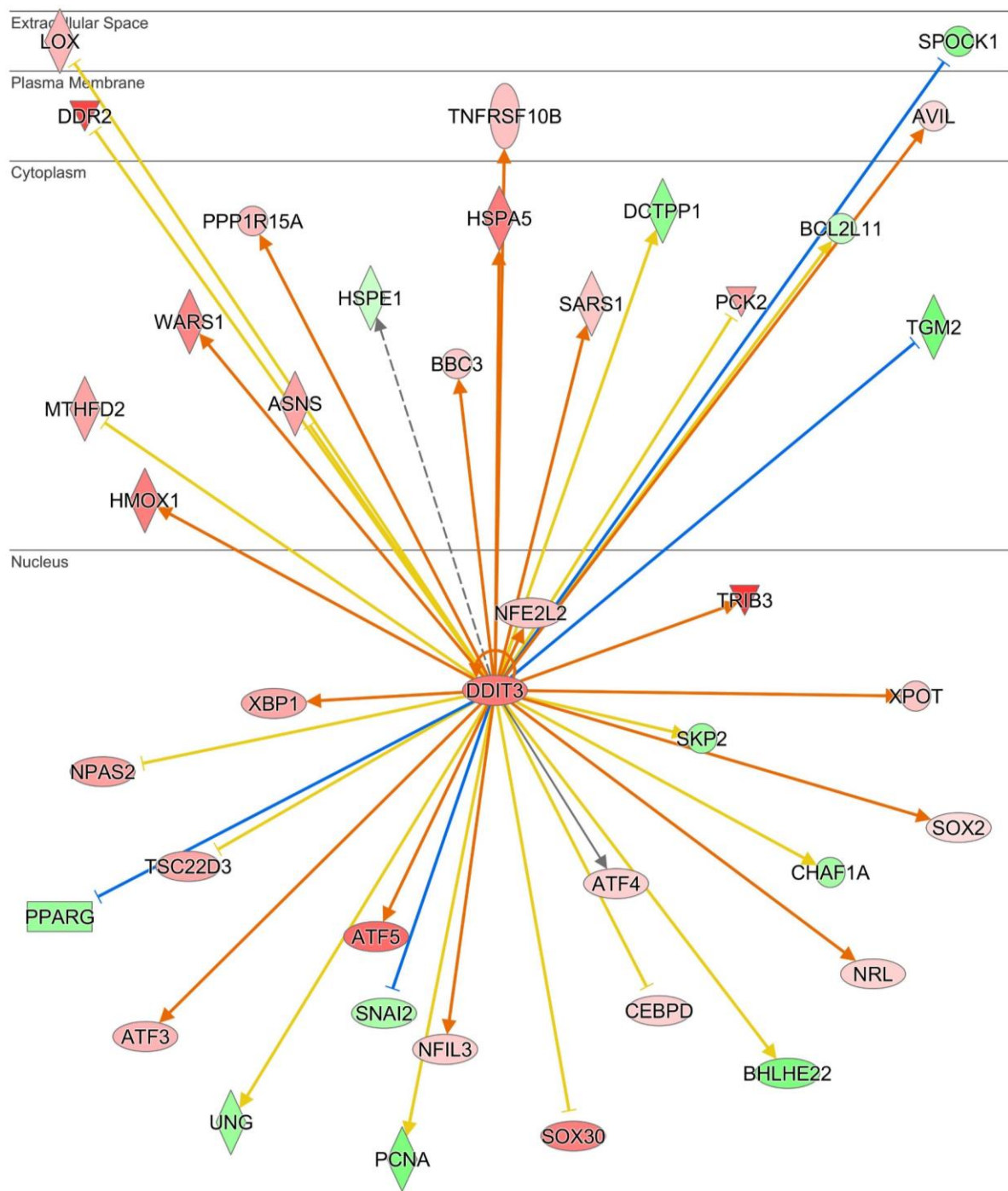

**Figure S1. *DDIT3* (*CHOP*) gene network showing its direct downstream DE targets.**

Supplement: Supplementary file 1 [file genes-15-00569-s001.zip › Figure S1.pdf]
